# Using nominal group technique to compare patients’ and clinicians’ perspectives on symptoms in multiple myeloma to inform the development of a self management tool for patients with relapsed myeloma

**DOI:** 10.12688/hrbopenres.12863.2

**Published:** 2021-02-16

**Authors:** Orlaith Cormican, Maura Dowling

**Affiliations:** 1 Cancer Clinical Trials Unit, HRB Clinical Research Facility, National University of Ireland Galway, National University of Ireland, Galway, Galway, Ireland; 2 School of Nursing and Midwifery, National University of Ireland, Galway, Galway, Ireland

**Keywords:** Nominal Group Technique, Relapsed Myeloma, Self Management, Patient Priorities, Healthcare app, Chronic illness management

## Abstract

**Background:** The nominal group technique (NGT) allows stakeholders to directly generate items for needs assessment. The objective was to demonstrate the use of NGT to inform the development of a healthcare app in patients with relapsed myeloma. Healthcare professionals with experience in the care of patients with relapsed/refractory myeloma were invited to participate.

**Methods:** One NGT group was conducted. In the group, health care professionals working in haematology were asked to vote anonymously in order of highest priority, on symptoms previously highlighted by relapsed/refractory myeloma patients in four focus groups.

**Results:** A total of 18 healthcare professionals working in the area of haematology participated in the NGT discussion; consultants (n=6), haematology registrars (n=2), specialist nurses [Advanced Nurse Practitioner/Clinical Nurse Specialist] (haematology) (n=3), staff nurse (n=1), and “other” health care professionals (n=6). Participants ranged in experience of working with myeloma patients from 2 years to over 27 years. The symptoms voted in highest priority were: Pain, Fatigue, Peripheral Neuropathy, Infection Risk and Steroid Induced Side Effects.

**Conclusions:** The NGT was an efficient method for obtaining information to inform a healthcare app.

## Introduction

Patient participation and engagement in research for the development of health research priorities has increased in importance more recently. The Irish health care system has supported this through the development of patient and public involvement in research. Involving patients is particularly important as their views may often differ from health care professionals and current evidence based practice, and their involvement can provide valuable insights. In addition, patient involvement provides opportunities for increased awareness of research trends, and communicating issues of importance to patients and their families in relation to their needs ^1^. Moreover, patient and health care professional partnerships in research can improve the patient’s health and can benefit the healthcare system generally.

In the study discussed here, the population were patients with relapsed multiple myeloma (MM). MM accounts for 2% of all cancers and is the 2 ^nd^ most common hematological malignancy ^2^. MM is a B cell malignancy resulting in uncontrolled production of plasma cells. These plasma cells overproduce immunoglobulins (heavy and light-chain M-proteins) known as a paraprotein, which build up in the bone marrow and often enter adjacent bone. The cause of MM is unknown; however it is known that the production of paraprotein in MM is associated with specific chromosomal abnormalities (deletions or translations). Almost all patients with MM will relapse, with each remission duration reducing as the number of treatment regimens increase ^3^. Patients with relapsed or refractory disease are defined as those who, having achieved minor response or better, relapse and then progress while on salvage therapy or experience progression within 60 days of their last therapy ^4^.

Patients with MM are living longer resulting in its treatment as a chronic disease rather than a terminal illness. Chronic diseases are life changing and increasingly patients with MM are living with long term symptoms as a result of treatment toxicities and side effects. Symptoms have been acknowledged as a hindrance to quality of life as well as having significant effects on emotional status, activity and participation in life ^5^.

In order for MM to be managed as a chronic condition patients need to be treated holistically, addressing not only their diagnosis but the symptoms and toxicities associated with treatment ^6^. MM patients also need to be empowered to take control of their condition enabling them to take control of their health ^7^. Evidence suggests that by helping patients to self-manage, the risk of adverse events and hospital admissions are reduced and patients’ quality of life is improved ^8^.

Treatment-specific clinically focused assessment tools can be useful for optimising therapy and implementing supportive care strategies (e.g., growth factors, transfusion support, intravenous hydration, bisphosphonates, antiviral therapies) to manage treatment-related symptoms. Patient education opportunities also arise when patients are assessed resulting in a heightened awareness and encouraging prompt reporting of symptoms and side effects. When education is approached efficiently it can significantly reduce the severity of symptoms and improve overall quality of life ^9^.

We report here on phase 2 (nominal group technique (NGT)) of a study aimed to develop a symptom management tool for patients with relapsed myeloma. The NGT approach was used to determine a consensus on the top 5 symptoms to include in the development of a symptom management tool. In the context of consensus methods, experts are those individuals who have knowledge about the topic of concern and it is a useful method to explore stakeholder views ^10^. In this case the experts were the healthcare professionals involved in the care of these patients. 

Modified NGT is a consensus planning tool that helps to prioritise issues ^11^. This technique was devised by Delbecq and Vandeven (1971) ^12^ and was chosen as the basis of our consensus as it preserved anonymity as well as fostering an exchange of opinions among multiple stakeholders in a non-threatening environment where balanced individual opinions are prioritised through a democratic method ^12^. In addition, the nominal group technique allows a comparison to be made between the healthcare consumer (i.e. the patient) and healthcare professionals but also ensures patient centred healthcare. This approach has proven popular in seeking patients’ involvement ^13, 14^.

While NGT is essentially a qualitative technique, its product is usually presented quantitatively by ranking the items produced by that group ^15^.

Advantages of the NGT include; ensuring dominant participants avoid control in a group setting, avoiding “quick” decision making, enabling the measurement of the importance of ideas/items to individuals and ensuring group cohesiveness ^15^. While NGT is capable of advancing knowledge in areas where conventional research evidence is lacking, it should not be viewed as a substitute for scientific methods of proven validity ^15^. 

In recent years, the lack of definitive studies on the effectiveness and appropriateness of health care interventions and policies has led to the increasing use of consensus methods ^15^. By including healthcare professionals in this exercise, a multi stakeholder approach is gained and this leads to the co production of health services research between patients and healthcare professionals, addressing the gap that often exists. It is also known that medical tools developed with clinician input have greater clinical application and relevance ^16^

## Methods

### Ethical considerations

Ethical approval was sought and granted by the Research Ethics Committee in National University of Ireland Galway (Approval Number 16-May-05). All health care professionals agreed to partake by consenting to complete the voting card anonymously (Supplementary File 1).

The nominal group technique employed in this paper was part of a 3 phase project for the development of a symptom management tool for patients with relapsed myeloma. Phase 1 involved focus groups with patients and their carers, phase 2 is discussed in this paper and phase 3 led to the development and pilot of a symptom management tool for patients with relapsed/refractory myeloma ^17^.

### Previous focus group – phase 1

We conducted four focus groups with relapsed multiple myeloma patients in various locations across Ireland ( Table 1). Some carers also attended the focus groups with their relatives. The findings of these focus groups are discussed elsewhere ^18^. We chose this method for phase 1 as we wanted to gain a description of the lived experience of symptom burden for this population and what self care strategies patients were already using.

**Table 1.** Patient demographic characteristics of focus groups. Population of relapsed multiple myeloma patients who participated in Phase 1 of the Research Project.

Participant (Myeloma patients) Number: 15	
Age	
18–45 years	0
46–55 years	1
56–65 years	8
66–80 years	6
80 + years	0
Gender	
Male	9
Female	6
Length of Diagnosis	
< 2 years	0
2–5 years	7
5–10 years	2
>10 years	5
Unknown	1
Marital Status:	
Single (never married)	1
Married	11
Separated/Divorced	0
Widowed	2
Unknown	1
Highest Educational Level	
Completed Primary School	0
Completed Secondary School	6
Attended Some Secondary School	5
Completed Third Level Education	4
Employment Status	
Employed	2
Unemployed	2
Retired	11
Supportive Measures Used	
Emotional/Psychological Support	1
Financial Support	2
Transportation Services	1
Support Groups	4
Internet Forums	2
Complementary Therapies	1
Charity Funded Organisations	2
Cancer Nurse Line	1
Nothing	9
Number of patients currently on treatment: 9	
Number of patients who know what treatment they are on: 6	
Number of patients who know how many lines of treatment they have received: 8	

At the focus groups, participants were asked to discuss their experience of common symptoms and adverse events and how these impacted on their quality of life. Patients’ experience of symptom management and what improvements could be made to symptom management were also explored as well as asking patients for their opinions on a self-management tool ^18^. All interviews were audio-recorded and transcribed and then analysed using thematic analysis, revealing 12 common symptoms within the patient experience.

### Modified NGT – phase 2

Modified NGT was subsequently employed, with a panel of health care professionals attending a local haematology seminar. The seminar takes place every quarter and health care professionals working within the area of haematology and with experience of caring for MM patients attend. Using NGT, we wished to identify the top 5 priority symptoms to be included in a symptom management tool. Therefore participants at the seminar were asked to vote (using anonymous voting cards) in order of priority ^1– 5^, what they deemed to be of highest priority symptoms for attention in the care of patients with relapsed myeloma (Supplementary File 1). Due to time and the feasibility of the project we chose to include only the 5 most prioritised symptoms that were considered burdensome from a patient and healthcare professional viewpoint.

### Recruitment

Recruitment of health care professionals was via communication channels of a regional haematology group who meet at set times over a calendar year. The chair of the haematology seminar informs health care professionals about the upcoming meetings and agenda via a group email. Following discussion with the chair, an email was sent to all members describing the research being undertaken, its aims and expected outcomes. Members were also informed about the intention to present phase 1 of this research project at the upcoming haematology seminar and the plan to conduct the modified nominal group technique following same. It was emphasised that partaking in the exercise would be voluntary and consent would be obtained by agreeing to fill out the voting card. See Supplementary File 1.

Initially one researcher presented the findings at the haematology seminar from phase 1 of the research project “Symptom management through self-management: Improving the outcomes of patients with relapsed myeloma.” This presentation included the background, progress and findings of phase 1 of the project as well as an explanation of the overall aim of the project, i.e. to develop a symptom management tool for patients. In addition the symptoms that were ranked as burdensome and explored in detail by patients were presented to the healthcare professionals (12 in total, Table 2).

**Table 2.** Votes from the modified group technique.

Symptoms/Side Effects	Number of Votes	Top 5 Highest Priority
Steroid Induced Side Effects	**11**	**5**
Anxiety	**5**	
Depression	**4**	
Fatigue	**15**	**2**
Peripheral Neuropathy	**13**	**3**
Infection Risk	**12**	**4**
Cytopenia	**5**	
Pain	**16**	**1**
Dental Issues	**1**	
Muscle Cramps	**0**	
Decreased Mobility	**5**	
GI Issues	**3**	

Health care professionals consented to participate in the NGT by way of agreeing to complete the questionnaire as well as the inclusion of a consent statement, to be completed before proceeding with the voting. A total of 18 health care professionals agreed to partake in the study.

Voting cards were distributed by the 2 ^nd^ author post-presentation and participants were asked to vote anonymously on what they perceived to be the five most burdensome symptoms for patients (Supplementary File 1).

Generally the Nominal Group technique involves a process of: “(i) silent generation of ideas by each individual, (ii) round-robin recording of ideas, (iii) structured and time-limited discussion of ideas, and (iv) selection and ranking of ideas (voting)” In this case the generation of ideas had already been obtained from the results of the focus group with patients and we did not seek engagement from the participants in a round robin recording of ideas ^19^. Following the presentation on the findings from phase 1 of the project, a group discussion took place on the meaning of the findings and then health care professionals were asked to vote about what they believed to be of most importance. Our end goal was to prioritise the symptoms based on data which had already been completed therefore we did not conduct the silent generation of ideas by individuals or the round robin recording of ideas.

## Results

In total, 18 health care professionals participated in the modified NGT; 6 Consultants, 2 haematology registrars, 3 specialist nurses [Advanced Nurse Practitioner/Clinical Nurse Specialist] (haematology), 1 staff nurse and 6 “other” health care professionals. Participants ranged in experience of working with myeloma patients from 2 years to greater than 27 years.

Our aim was to include the top 5 priorities for inclusion in the app; therefore the results of the voting cards were reviewed to determine which symptoms received the most votes and the ranking of the votes. Of the 12 symptoms identified by patients in the focus groups, pain was the top symptom voted by the HC professionals as essential for inclusion in a patient app. The symptom voted second was fatigue with peripheral neuropathy, infection risk and steroid induced side effects following as the symptoms that were of highest priority in the management of patients with relapsed or refractory multiple myeloma ( Table 2).

## Discussion

This modified nominal group process provided useful information from health care professionals for the purposes of gaining consensus on what they perceived to be symptom priority for relapsed myeloma patients. Interestingly, there were similar findings between patients and health care professionals in relation to the most challenging symptoms experienced.

Fatigue and steroid induced side effects were considered two of the symptoms of highest priority for inclusion in the symptom management app. Fatigue is described in the literature as one of the most common distressing symptoms in MM. Associated causes include anaemia, pain, mood disturbance, diminished strength, diminished endurance, decreased sleep efficiency at night and advanced disease. It has also been reported that fatigue has the greatest negative effect on physical functioning in those with a diagnosis of MM. Despite this ongoing symptom burden, treatment remains episodic and self-management programmes have been limited for those on lifelong treatment ^20^. Patients have already described some self-care strategies used such as resting times, distraction techniques “mind over matter”, exercise and “walking off the fatigue.” ^18^

Steroid induced side effects are often acknowledged in the development of fatigue as a long term side effect in patients with relapsed myeloma. Despite the significant impact steroids have on neuropsychiatric function and health related quality of life, it remains a useful therapy in the control of MM through improving overall patient response as well as progression free survival. Their mechanism is complex; however they are thought to modify the body’s immune system and produce powerful anti-inflammatory properties. In contrast to their ability to help in the control of MM they are known to cause intense adverse effects including elevated blood sugars, increased risk of infection, mood swings and insomnia, which are often long term and not widely described or documented despite the common use of steroids ^21^. Phase 1 of our study iterated these adverse events with patients describing hyperactivity, the “let down” effect and excessive diaphoresis as having a significant impact on their health related quality of life ^18^. Patients were very aware of the associated side effects of steroids and reported the side effects as out of control at times. However it has been noted that patients appreciate the impact steroids can have on their disease response so they often fail to report side effects affecting their health related quality of life ^18^. As such, it is of paramount importance that health care professionals educate patients and their caregivers on the importance of reporting adverse effects and thus improve patient outcomes. Education and patient involvement could improve the longer term outcomes of relapsed MM patients on steroids ^18^.

The technique that we used involved a modification of the NGT. Generally there are at least 5–6 steps involved in the process; however we chose a two-step process which included two different groups of participants (patients and health care professionals). Using this technique ensured that individuals did not feel pressure to conform based on perceived status within the group, with all health care professionals voting anonymously. In addition it allowed the stakeholders to prioritise the symptoms through a democratic process ^22^.

This approach has however a number of limitations. Focus group discussions with health care professionals would have provided a more comprehensive picture of symptom management for relapsed MM patients. Using just the modified NGT was a very blunt approach which provided conclusions without context. Further data would most likely have been generated if we had not modified the NGT process.

Nominal groups often request participants to record ideas independently and in private, then sharing, listing and discussing the ideas, and finally judging or voting on the ideas independently. It is usual for participants to be asked to identify issues before attending the NGT session, however in this case the issues were already identified ^13^.

## Conclusion

NGT offers opportunity to involve both patients and health care professionals to be involved in healthcare improvement and design. Research prioritisation now needs to focus on both patient and health care professional viewpoints to ensure a comprehensive approach to care. NGT offers potential in gaining an insight to health care professionals’ priorities for patients and addressing the gap that is often described between health care professionals and patient values. The findings in this work provide opportunity to address the gap that often is described between patient and health care professional values.

## Data availability

### Underlying data

All data underlying the results are available as part of the article and no additional source data are required.

### Extended data

Figshare: Supplementary_File_1 Voting Card

https://doi.org/10.6084/m9.figshare.13954283.v1 ^23^

This project contains the following underlying data:

- Supplementary_File_1 Voting Card.docx (Using NGT, we wished to identify the top 5 priority symptoms as voted by health care professionals to be included in a symptom management tool for patients with relapsed/refractory myeloma. Participants at the seminar were asked to vote (using anonymous voting cards) in order of 1–5 , what they deemed to be of highest priority symptoms for attention in the care of patients with relapsed myeloma (Supplementary File 1).)

Data are available under the terms of the Creative Commons Zero “No rights reserved” data waiver (CC0 1.0 Public domain dedication).
