# Supplementary material for: Using nominal group technique to compare patients’ and clinicians’ perspectives on symptoms in multiple myeloma to inform the development of a self management tool for patients with relapsed myeloma
Source: HRB Open Res. 2024 Oct 21;1:21. Originally published 2018 Aug 31. [Version 3] doi: 10.12688/hrbopenres.12863.3 (PMC11372347; doi:10.12688/hrbopenres.12863.3)
Supplement: Peer review history

# Reviewer report: Reviewer response for version 2

**DOI:** 10.21956/hrbopenres.14348.r41786

**Published:** 2024-09-03

**Authors:** Debra Howell

**Affiliations:** 1 University of York, York, UK

Thank you for asking me to review this revised paper. The subject is of interest. Comments are as follows:

 It was unclear if the purpose of the assessment tool was to help people manage their own symptoms (would this be possible for some of the issues), or so they can highlight/discuss them with clinical staff.

 Regarding 'symptom priority', was this in terms of the issues that were most serious, or that had most impact on quality of life for patients, or were most inconvenient? This was unclear and needs to be explained.

 I wasn't sure when data were collected as there is no date.

 The characteristics of patients from the focus groups are shown in Table 1, not participants in the NGT. For this paper, I would have liked to know if they were representative of the population of myeloma patients. 

 I would have like to know if the 5 priorities given by the clinicians matched those of patients? Surely that information is relevant and would need further exploration for the app. This is linked to the definition of 'symptom priority' - as noted above.

 The sample size for what is essentially a ranking exercise is very small, which raises issues of generalisability.

Is the work clearly and accurately presented and does it cite the current literature?

Partly

If applicable, is the statistical analysis and its interpretation appropriate?

Not applicable

Are all the source data underlying the results available to ensure full reproducibility?

Yes

Is the study design appropriate and is the work technically sound?

Partly

Are the conclusions drawn adequately supported by the results?

Partly

Are sufficient details of methods and analysis provided to allow replication by others?

Yes

Reviewer Expertise:

25 years examining blood cancer patient experiences, including  symptoms in the context of myeloma.

I confirm that I have read this submission and believe that I have an appropriate level of expertise to confirm that it is of an acceptable scientific standard, however I have significant reservations, as outlined above.

# Response

**Published:** 2024-10-16

**Authors:** Orlaith Cormican

**Affiliations:** National University of Ireland, Galway, Ireland

Hi Debra,

 Thank you for completing this review.

 Please see comments in relation to your review (included in italics)

 It was unclear if the purpose of the assessment tool was to help people manage their own symptoms (would this be possible for some of the issues), or so they can highlight/discuss them with clinical staff.

*The purpose of the assessment tool was to help people manage their own symptoms and to highlight or discuss them with staff. I will make the necessary changes to the article so there is clarity.*

 Regarding 'symptom priority', was this in terms of the issues that were most serious, or that had most impact on quality of life for patients, or were most inconvenient? This was unclear and needs to be explained.

*The symptoms were to be prioritised in terms of what had the most impact on patients quality of life and their ability to manage them.*

 I wasn't sure when data were collected as there is no date.

*The research was conducted in 2018.*

 The characteristics of patients from the focus groups are shown in Table 1, not participants in the NGT. For this paper, I would have liked to know if they were representative of the population of myeloma patients. 

*The participants in the NGT were clinicians - their characteristics are described under the results section.*

 I would have like to know if the 5 priorities given by the clinicians matched those of patients? Surely that information is relevant and would need further exploration for the app. This is linked to the definition of 'symptom priority' - as noted above.

*The 12 symptoms initially included were described by the patients in the focus groups. We did not explore what was of the highest priority for the patients after they were identified by the clinicians but this could be considered in future work going forward.*

 The sample size for what is essentially a ranking exercise is very small, which raises issues of generalisability.

*This is correct - I will include this as a limitation.*

*Many thanks,*

*Orlaith Cormican.*

# Reviewer report: Reviewer response for version 2

**DOI:** 10.21956/hrbopenres.14348.r28915

**Published:** 2021-03-15

**Authors:** Angela Stover

**Affiliations:** 1 Lineberger Comprehensive Cancer Center, University of North Carolina at Chapel Hill, Chapel Hill, USA; 2 Department of Health Policy and Management, University of North Carolina at Chapel Hill, Chapel Hill, USA

Thank you for revising your manuscript. It is clear now and flows well. Reviewer concerns have been addressed.

Is the work clearly and accurately presented and does it cite the current literature?

Partly

If applicable, is the statistical analysis and its interpretation appropriate?

Not applicable

Are all the source data underlying the results available to ensure full reproducibility?

Yes

Is the study design appropriate and is the work technically sound?

Partly

Are the conclusions drawn adequately supported by the results?

No

Are sufficient details of methods and analysis provided to allow replication by others?

Partly

Reviewer Expertise:

Patient reported outcome (PRO) development, symptom science, mixed methods, oncology, implementation science.

I confirm that I have read this submission and believe that I have an appropriate level of expertise to confirm that it is of an acceptable scientific standard.

# Reviewer report: Reviewer response for version 1

**DOI:** 10.21956/hrbopenres.13930.r28231

**Published:** 2020-11-16

**Authors:** Angela Stover

**Affiliations:** 1 Lineberger Comprehensive Cancer Center, University of North Carolina at Chapel Hill, Chapel Hill, USA; 2 Department of Health Policy and Management, University of North Carolina at Chapel Hill, Chapel Hill, USA

This is an observational study where 18 healthcare professionals anonymously voted once on their top 5 MM symptoms. MM is an area ripe for interventions to detect and manage symptoms given the high symptom burden.

 Title is misleading. Paper is not about development of a self-management app, but asking healthcare professionals for their perception of what the top 5 MM symptoms are. It sounds like it is step one of a long development process.

 Introduction starts with the importance of involving patients in generating symptom domains, but the patient focus group was published elsewhere. The current paper is about the clinician perspective so the intro would make more sense with that focus. Another option is to reframe the paper as using NGT to compare patient and clinician perspectives on MM symptoms to include in an app. The intro would still need to include why the clinician perspective is important.

 The paper could be tightened and strengthened throughout.

 The introduction would be strengthened by explaining what the Nominal Group Technique is and why it is relevant here. The conclusion in the abstract is that NGT was useful so setting up the paper with a discussion of NGT is warranted. The NGT paragraph on pg. 4 could be moved to the intro and expanded.

 Previous focus group: what were the 12 common symptoms from the patient experience?

 Modified NGT. Please explain what the modification is and why it is needed. 

 Please provide a justification for why clinicians were asked to generate only 5 symptoms. MM has many possible symptoms. Patients generated 12 symptoms in a previous study.

 Calling the methods a focus group is misleading throughout. Clinicians individually voted (anonymously) on their top 5 symptoms. 

 Table 1 includes the patient characteristics... but this study is not about the patient perspective unless you reframe the paper. 

 Is Table 2 from the patient or clinician perspective? It is labeled as both in the text. Labeling the column headers better would help reduce confusion.

Is the work clearly and accurately presented and does it cite the current literature?

Partly

If applicable, is the statistical analysis and its interpretation appropriate?

Not applicable

Are all the source data underlying the results available to ensure full reproducibility?

Yes

Is the study design appropriate and is the work technically sound?

Partly

Are the conclusions drawn adequately supported by the results?

No

Are sufficient details of methods and analysis provided to allow replication by others?

Partly

Reviewer Expertise:

Patient reported outcome (PRO) development, symptom science, mixed methods, oncology, implementation science.

I confirm that I have read this submission and believe that I have an appropriate level of expertise to state that I do not consider it to be of an acceptable scientific standard, for reasons outlined above.

# Response

**Published:** 2020-12-14

**Authors:** Orlaith Cormican

**Affiliations:** National University of Ireland, Galway, Ireland

Thank you for your comments and taking the time to review our article. Please see below our comments in bold.

- Title is misleading. Paper is not about development of a self-management app, but asking healthcare professionals for their perception of what the top 5 MM symptoms are. It sounds like it is step one of a long development process. **We have changed the title and are hopeful that this reflects what the article contains.**
- Introduction starts with the importance of involving patients in generating symptom domains, but the patient focus group was published elsewhere. The current paper is about the clinician perspective so the intro would make more sense with that focus. Another option is to reframe the paper as using NGT to compare patient and clinician perspectives on MM symptoms to include in an app. The intro would still need to include why the clinician perspective is important. **More information has been added to the introduction which will hopefully clarify this.**
- The introduction would be strengthened by explaining what the Nominal Group Technique is and why it is relevant here. The conclusion in the abstract is that NGT was useful so setting up the paper with a discussion of NGT is warranted. The NGT paragraph on pg. 4 could be moved to the intro and expanded. **We have added to the introduction to include more information about the Nominal Group technique and have taken your suggestion about the paragraph on page 4 and moved it.**
- Previous focus group: what were the 12 common symptoms from the patient experience? **These are listed in Table 2.**
- Modified NGT. Please explain what the modification is and why it is needed. **Same has been added.**
- Please provide a justification for why clinicians were asked to generate only 5 symptoms. MM has many possible symptoms. Patients generated 12 symptoms in a previous study. **Justification now added.**
- Calling the methods a focus group is misleading throughout. Clinicians individually voted (anonymously) on their top 5 symptoms. **The methods are not called a focus group. The focus group was phase 1 of a larger project which led to phase 2 of this project. I have included further clarification in the methods section.**
- Table 1 includes the patient characteristics... but this study is not about the patient perspective unless you reframe the paper. **We thought it was important to include table 1 to give background to the project and the patients that partook in phase 1 that led on to phase 2; that is the modified NGT.**
- Is Table 2 from the patient or clinician perspective? It is labeled as both in the text. Labeling the column headers better would help reduce confusion. **We have included a header which should make this table clearer. **

# Reviewer report: Reviewer response for version 1

**DOI:** 10.21956/hrbopenres.13930.r26415

**Published:** 2019-05-16

**Authors:** Peter Cudd

**Affiliations:** 1 CATCH, University of Sheffield, Sheffield, UK

The article addresses an important need to involve users – patients and professionals of proposed technology in its specification – in this case a proposed app for better management of symptoms of relapsed myeloma. There are a number of issues that would improve the article, those that are essential are asterisked.

 The Nominal Group technique appears to be extensively used in myeloma research, the cited references and introduction could better reflect this. For example the closing paragraph of the Introduction could state that ‘the approach was to determine a consensus using the widely used NGT [refs here].*

 The study design is one amongst many possibilities and some justification is given for its selection. However the authors failed to describe the where and how of the recruitment of the professionals for replication and also no consideration is provided for how homogeneous the context is, i.e. is it reasonable to assume there is widely shared symptoms/issues of concern, that staff in one service is going to have similar profiles of views, that different types of professionals will have similar views. The latter becomes important for interpretation of findings given the bias in recruits of doctors over nursing staff in their sample (which also isn’t discussed).*

 The authors have found that the method was useful but several questions arise that it would have explained. Why patients and then service staff in the two phases, why not the other way round, or if able to do both have some involved in both phases (possibly separated) and possibly something that would strengthen the findings to different recruits in the implied 4 groups in the latter case? It may be convenience and/or feasibility with project resources or indeed the intended use of the list of the top 5.* Just what value can the reader place on the particular findings they have – so that some judgement can be made about applying the technique elsewhere. The authors have given a vague description of what is next – but they should make clear how what they have done here fits in with that.*

 In the Methods section we are led to believe that the ‘Modified NGT – phase 2’ is the focus of the article. But when you read the conclusions you find that you could make the inference that Phase 1 is part of the NGT. The comments about patients and professionals are able to take part using this technique is generally true and the opening sentences are statements that could be made from other articles from the field (and yet they are not cited to support this conclusion). If these were added then a more appropriate positioning of the findings of the authors work could be made by starting a sentence to follow which states the findings in this study confirm user participation.*

 I suggest the final sentence of the Conclusions is moderated somewhat concerning the gap, e.g. it is possible for NGT approach to make a particular contribution to the gap between professional and patient values, that contribution very much depends on how it is implemented and biases of those involved (including the researchers).

 It may be useful for the authors to consider that the patients will be the ones who will be asked to routinely adhere to using the app and yet the professionals have to believe in it to prescribe/recommend it.

Is the work clearly and accurately presented and does it cite the current literature?

Partly

If applicable, is the statistical analysis and its interpretation appropriate?

Not applicable

Are all the source data underlying the results available to ensure full reproducibility?

Yes

Is the study design appropriate and is the work technically sound?

Partly

Are the conclusions drawn adequately supported by the results?

Yes

Are sufficient details of methods and analysis provided to allow replication by others?

Partly

Reviewer Expertise:

Digital technology and service innovation through user participation, Adult persistent fatigue (across diseases).

I confirm that I have read this submission and believe that I have an appropriate level of expertise to confirm that it is of an acceptable scientific standard, however I have significant reservations, as outlined above.

# Response

**Published:** 2020-12-14

**Authors:** Orlaith Cormican

**Affiliations:** National University of Ireland, Galway, Ireland

Thank you for your comments and taking the time to review our article. Please see our responses below in bold.

- The Nominal Group technique appears to be extensively used in myeloma research, the cited references and introduction could better reflect this. For example the closing paragraph of the Introduction could state that ‘the approach was to determine a consensus using the widely used NGT [refs here].* **We have added further clarification to same which better reflects this.**
- The study design is one amongst many possibilities and some justification is given for its selection. However the authors failed to describe the where and how of the recruitment of the professionals for replication and also no consideration is provided for how homogeneous the context is, i.e. is it reasonable to assume there is widely shared symptoms/issues of concern, that staff in one service is going to have similar profiles of views, that different types of professionals will have similar views. The latter becomes important for interpretation of findings given the bias in recruits of doctors over nursing staff in their sample (which also isn’t discussed).* **We have included detail about recruitment. We believe that the sample is homogenous given that the health care professionals all work in the same area, i.e haematology.** **Regarding your comment “is it reasonable to assume there is widely shared symptoms/issues of concern, that staff in one service is going to have similar profiles of views, that different types of professionals will have similar views” The results in Table 2 reflect how health care professionals voted, therefore we don’t consider this an assumption. Regarding your comment on bias: We have included further information regarding bias in terms of decision making, however the sample was chosen at random and we did not know how many doctors would be recruited over nursing staff.**
- The authors have found that the method was useful but several questions arise that it would have explained. Why patients and then service staff in the two phases, why not the other way round, or if able to do both have some involved in both phases (possibly separated) and possibly something that would strengthen the findings to different recruits in the implied 4 groups in the latter case? **We have included the explanation for this at the end of the introduction.** It may be convenience and/or feasibility with project resources or indeed the intended use of the list of the top 5.* Just what value can the reader place on the particular findings they have – so that some judgement can be made about applying the technique elsewhere. The authors have given a vague description of what is next – but they should make clear how what they have done here fits in with that.*More clarification has been added about why the top 5 symptoms were to be included in the tool. **I don’t think it is necessary to include further detail about phase 3 of the project.**
- In the Methods section we are led to believe that the ‘Modified NGT – phase 2’ is the focus of the article. But when you read the conclusions you find that you could make the inference that Phase 1 is part of the NGT. The comments about patients and professionals are able to take part using this technique is generally true and the opening sentences are statements that could be made from other articles from the field (and yet they are not cited to support this conclusion). **We have removed some information from the conclusion so that the focus remains on the NGT,** If these were added then a more appropriate positioning of the findings of the authors work could be made by starting a sentence to follow which states the findings in this study confirm user participation.* **More information added to the conclusion**
- I suggest the final sentence of the Conclusions is moderated somewhat concerning the gap, e.g. it is possible for NGT approach to make a particular contribution to the gap between professional and patient values, that contribution very much depends on how it is implemented and biases of those involved (including the researchers). **All care was taken to reduce bias in phase 1 of the study and this is discussed elsewhere. I don’t see how bias is an issue here.**
-  It may be useful for the authors to consider that the patients will be the ones who will be asked to routinely adhere to using the app and yet the professionals have to believe in it to prescribe/recommend it. **This is discussed elsewhere.**
